# Umbilical cord mesenchymal stem cells relieve osteoarthritis in rats through immunoregulation and inhibition of chondrocyte apoptosis

**DOI:** 10.1038/s41598-023-42349-x

**Published:** 2023-09-11

**Authors:** Xin Pan, Xiongfeng Li, Ling Zhang, Feifei Wu, Qiang Zhang, Shasha Xu, Chengchun Shen, Jinfeng Liang, Ruolang Pan

**Affiliations:** 1 Institute for Cell-Based Drug Development of Zhejiang Province, S-Evans Biosciences, No. 181 Wuchang Road, Hangzhou, 311122 Zhejiang China; 2 Key Laboratory of Cell-Based Drug and Applied Technology Development in Zhejiang Province, Hangzhou, China; 3 Huzhou Basic and Clinical Translation of Orthopaedics Key Laboratory, Affiliated Huzhou Hospital, Huzhou, China; 4 Zhejiang Center for Drug & Cosmetic Evaluation, Hangzhou, 310012 China

**Keywords:** Drug discovery, Stem cells

## Abstract

This study aims to investigate the effectiveness of umbilical cord mesenchymal stem cells (UCMSCs) in treating osteoarthritis (OA). Sprague–Dawley rats were used in in vivo experiments and divided into four groups: normal, OA model, saline, and UCMSC-treated groups (n = 6). An OA model was established by injecting iodoacetic acid into the joint cavity. The results indicate that UCMSC transplantation significantly reduced joint surface and articular cartilage damage, and the levels of IL-1β, TNF-α, and MMP13 in the joint fluid were significantly reduced after UCMSC treatment. In vitro experiments showed that co-culturing UCMSCs and chondrocytes promoted the expression of aggrecan, COL2, SOX9, and BCL-2; downregulated the expression of BAX and BAD in chondrocytes; and promoted the expression of IL-10 and TGF-β1 in UCMSCs. Additionally, the supernatant of UCMSCs inhibited the expression of IL-1β and TNF-α in the articular cavity and promoted the expression of COL2 and aggrecan in vivo. These effects were impaired when IL-10 and TGF-β1 were removed. Collectively, UCMSC transplantation appears to improve joint pathology, reduce inflammatory factors, and decrease chondrocyte apoptosis, likely through the involvement of IL-10 and TGF-β1, thus providing a potential therapeutic option for patients with OA.

## Introduction

Osteoarthritis (OA) is a prevalent musculoskeletal disease caused by the degeneration of articular cartilage and subchondral bone^1–4^. This condition affects more than half of individuals over 65 years of age and is characterized by constant pain, stiffness, and disability^5^. OA is expected to become a major cause of pain and disability as the global population ages. The pathogenesis of OA involves a variety of complex mechanisms, involving genetic, mechanical, metabolic, and inflammatory factors. Accumulating evidence shows that inflammatory factors, abnormal chondrocyte apoptosis, and extracellular matrix degradation are associated with the pathogenesis of OA^6,7^. The conventional treatment of OA mainly involves pain management, including exercise, physical therapy, lifestyle changes, administration of analgesics, and joint replacement surgery^8,9^. However, these treatments only temporarily relieve symptoms rather than target the pathogenesis or reverse OA progression. Despite its effectiveness, joint replacement surgery is often accompanied by several disadvantages, including infection with exogenous implant material, a short life span of the implant or tissue substitute, and a lack of newly formed tissue and natural cartilage. Therefore, there is an urgent need to indentify new treatments for OA. Recent advances have highlighted the potential of mesenchymal stem cell (MSC)-based therapies for treating OA^10^.

MSCs are precursors of connective tissue cells. They possess excellent self-renewal abilities and have the potential to differentiate into various mesodermal and endo-ectodermal cells, including adipocytes, osteoblasts, and chondrocytes^11,12^. The main characteristics of MSCs, such as their effective implantation, long-term survival, and low risk of immune rejection due to the low surface expression levels of major histocompatibility complex (MHC) antigens, make them therapeutically useful in OA^13^. For instance, bone marrow-derived MSCs have been widely used in animal models and clinical cases to study their cartilage-formation potential for OA treatment^14^. Umbilical cord mesenchymal stem cells (UCMSCs) have shown promise in the treatment of various diseases, because they can maintain their stemness even after multiple passages and amplification^15,16^. UCMSCs are considered a superior option compared to bone marrow MSCs because of their exceptional cloning, proliferation, migration, and chondrogenic potential^17,18^. Furthermore, UCMSCs have several distinct advantages, including ethical considerations and easy access to discarded umbilical cords without causing pain^19,20^. Currently, UCMSCs are used to treat many diseases, such as liver diseases, arthritis, diabetes and its complications, systemic lupus erythematosus, heart diseases, brain injuries, cerebrovascular diseases, and respiratory system diseases, highlighting that they are a significant therapeutic breakthrough^21^. Many studies have shown that UCMSCs are well tolerated by patients and can safely and effectively alleviate the symptoms of rheumatoid arthritis^22,23^. However, to date, only a few reports have explored the application of UCMSCs in OA^24^, and the underlying mechanism remains unclear. Hence, more efforts are needed to uncover the function of UCMSCs in OA.

In this study, we explored the effect of UCMSCs transplantation in a rat model of iodoacetic-acid-induced OA using the Kellgren-Lawrence score, Pelletier score, histopathology, and the detection of inflammatory factors in the synovial fluid. In addition, we conducted in vitro co-culture experiments and injected UCMSC-derived supernatants in rats with OA to study the specific mechanisms. Our results showed that the administration of UCMSCs increased the expression levels of interleukin-10 (IL-10) and transforming growth factor beta 1 (TGFβ-1), promoted the expression of chondrocyte-related genes, and reduced the apoptosis rate of chondrocytes. Furthermore, UCMSC-derived supernatants and UCMSCs inhibited the expression of inflammatory factors and promoted the expression of chondrocyte-related genes. However, this effect was inhibited when IL-10 and TGFβ-1 were removed from the supernatant. Our study demonstrated that UCMSCs have excellent therapeutic effects in a rat model of OA by reducing chondrocyte apoptosis and protecting chondrocytes, potentially via the secretion of IL-10 and TGF-β1.

## Results

### X-ray imaging of the knee joint structure

**Figure 1:**
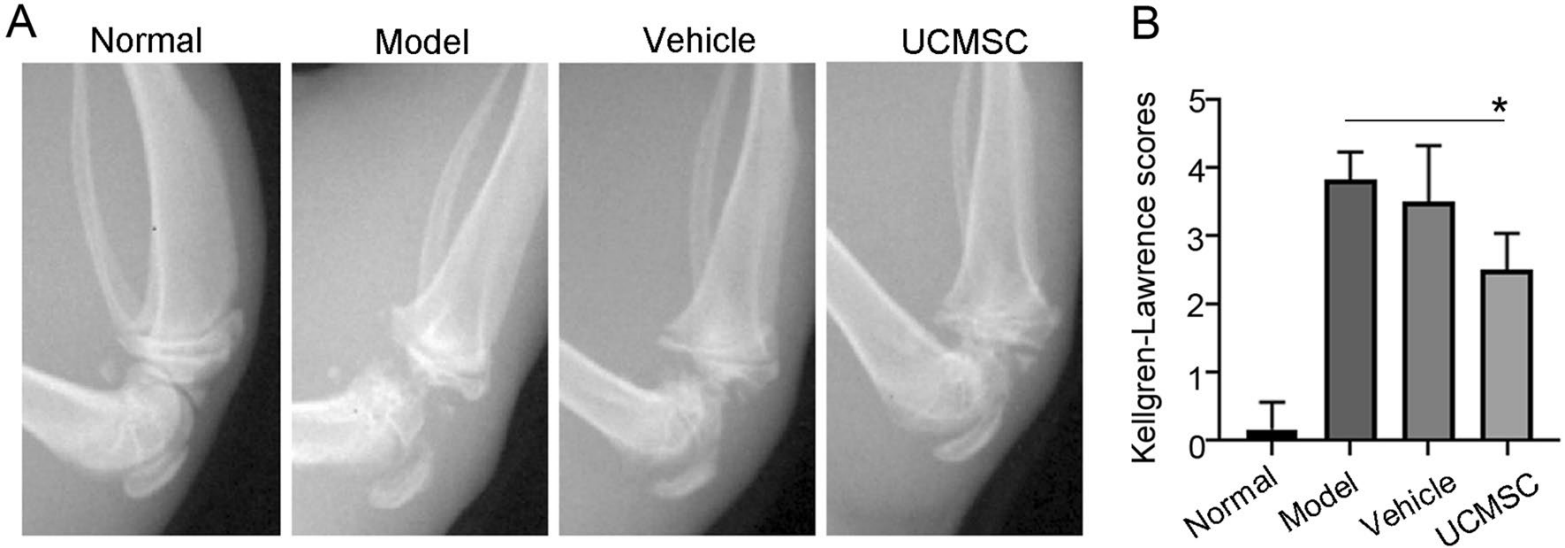
UCMSCs transplantation improved the knee joint structure of rats with OA. One week after the third UCMSC transplantation, X-rays were used to examine the structure of the rats’ knee joints. The OA model group had irregular articular surfaces, deformities, cartilage defects, tangential fissures, and obvious osteophytes. The articular surface of the UCMSC group was relatively flat. (**A**) Joint X-ray imaging. (**B**) Kellgren Lawrence score of joint X-ray imaging. Normal, normal group; Model, OA model group; Vehicle, OA model with saline injection; UCMSC, OA model with UCMSC transplantation, n = 6. *P < 0.05 analyzed by unpaired two-tailed t-test after normality and equal variance test using Shapiro–Wilk test and ANOVA plus Brown-Forsythe test in the GraphPad software.

UCMSCs were characterized (Supplementary Fig. 1) and injected into the joint cavities of the rats. After treatment, X-ray examination revealed that the knee joint surface of rats in the OA model group (n = 6) was uneven and deformed, exhibiting cartilage defects and apparent osteophyte formation, compared to the knee joint surface in rats in the normal group (n = 6; Fig. 1). We further observed that the articular surface was flatter in the UCMSC-treated group (n = 6) than the OA model group (*P* < 0.05), suggesting that UCMSC transplantation significantly improved the pathological score of the knee joint structure.

### Pathological changes in the articular surface

**Figure 2:**
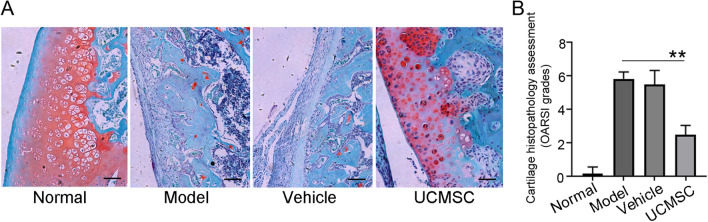
UCMSCs alleviated the pathological process of OA. (**A**) Safranin O staining of joint tissues. There was an increased number of cartilage cells that survived and thickened the cartilage surface in the UCMSC group compared with the OA model group. Scale bars = 100 µm. (**B**) OARSI classification of Safranin O staining. OARSI classification was used to assess the degree of pathological damage to articular cartilage. Normal, normal group; Model, OA model group; Vehicle, OA model with saline injection; UCMSC, OA model with UCMSC transplantation, n = 6. **P < 0.01 analyzed by unpaired two-tailed t-test after normality and equal variance test using Shapiro–Wilk test and ANOVA plus Brown-Forsythe test in the GraphPad software.

Paraffin sections were stained with safranin O and hematoxylin and eosin (H&E; Fig. 2A and Supplementary Fig. 2A). The results showed that the cartilage thickness was reduced in the OA group. UCMSC administration improved the pathology of rat knee joints to varying degrees. We observed a significant decrease in Osteoarthritis Research Society International (OARSI) scores in the UCMSC-treated group (*P* < 0.01; Fig. 2B and Supplementary Fig. 2B). These results suggest that UCMSCtransplantation can effectively repair the joint structure in rats with OA.

### Umbilical cord mesenchymal stem cells reduced the levels of inflammation-related factors in synovial fluid and protected chondrocytes

**Figure 3:**
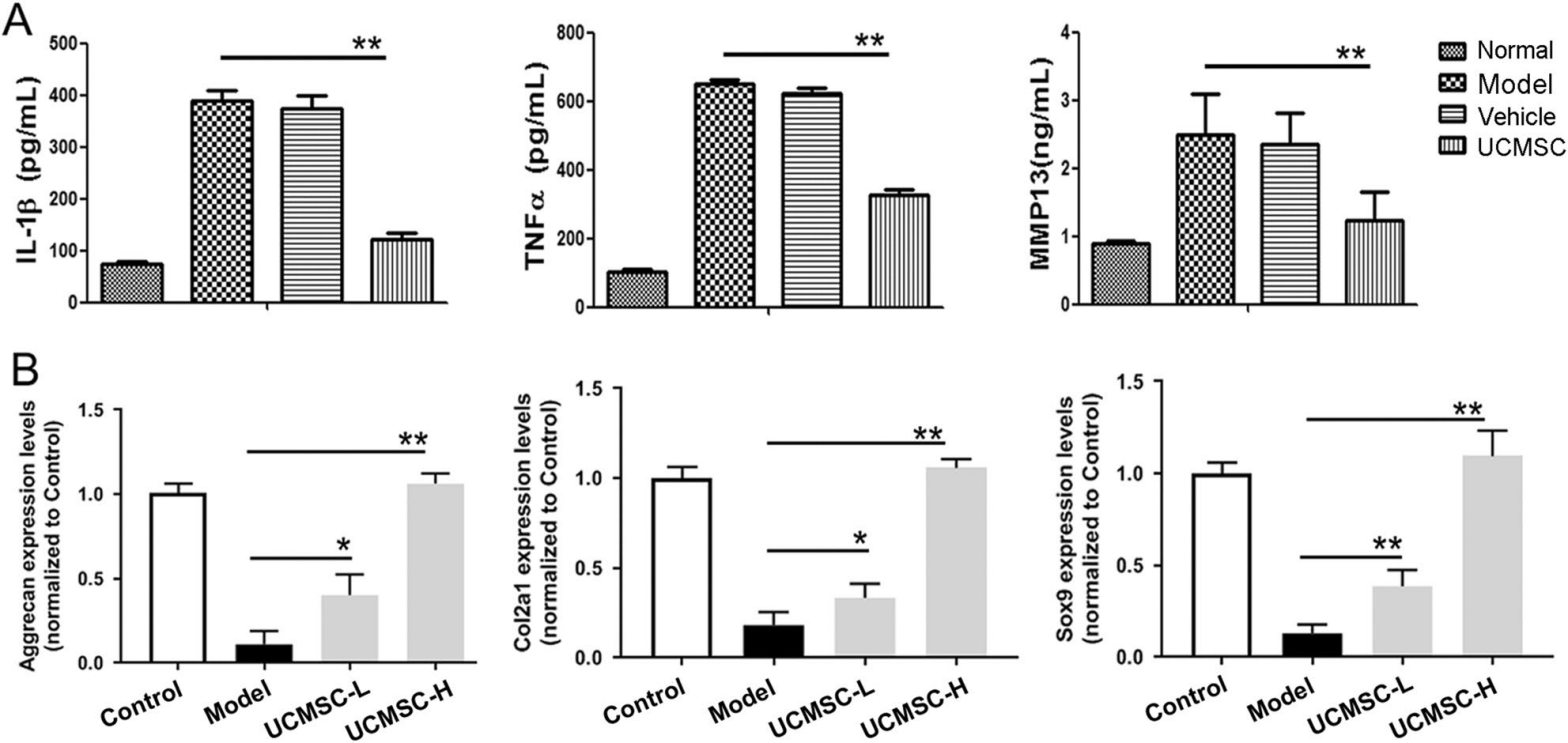
Inflammation inhibition and chondrocyte protection by UCMSCs. (**A**) Detection of inflammatory factors in synovial fluid. ELISAs were performed to detect IL-1β, TNF-α, and MMP13 in synovial fluid. The levels of these three factors decreased significantly in the UCMSC group compared with the model group, indicating that UCMSC transplantation inhibited the expression of key inflammatory factors in OA. Normal, normal group; Model, OA model group; Vehicle, OA model with saline injection; UCMSC, OA model with UCMSC transplantation. n = 6. (**B**) The effect of UCMSCs on the gene expression levels of *aggrecan*, *Col2,* and *Sox9* in chondrocytes induced by IL-β1. Chondrocytes co-cultured with UCMSCs and IL-β1 were collected, and RT-PCR was used to detect gene expression levels in chondrocytes. Treatment of chondrocytes with high (1 × 10^6^ cells) and low (0.5 × 10^6^ cells) doses of UCMSCs promoted the expression of *aggrecan*, *Col2*, and *Sox9* genes. Control, normal cultured rat chondrocytes; Model, rat chondrocytes pre-treated by 10 ng/mL IL-1β; UCMSC-L, rat chondrocytes pre-treated by 10 ng/mL IL-1β were co-cultured with 0.5 × 10^6^ UCMSCs; UCMSC-H, rat chondrocytes pre-treated by10ng/mL IL-1β were co-cultured with 1 × 10^6^ UCMSCs, n = 3. *P < 0.05, **P < 0.01 analyzed by unpaired two-tailed t-test after normality and equal variance test using Shapiro–Wilk test and ANOVA plus Brown–Forsythe test in the GraphPad software.

The key inflammatory factors, IL-1β and TNF-α, and articular cartilage matrix-degrading enzymes, such as MMP13, promote the occurrence of OA. We used enzyme-linked immunosorbent assays (ELISAs) to detect these proteins in the synovial fluid of rats to determine any changes in their levels during treatment with UCMSCs. We found that the levels of IL-1β, TNF-α, and MMP13 were significantly increased in the synovial fluid of rats in the OA model group (*P* < 0.01; Fig. 3A). However, the levels of all these factors were significantly decreased in the UCMSC-treated group (*P* < 0.01). These findings show that UCMSC transplantation can inhibit the expression of key inflammatory factors in OA.

SOX9 is an early transcription factor that plays essential roles in cartilage formation. As a downstream target protein of SOX9, COL2 is a crucial component of the cartilage matrix. In addition, aggrecan plays an important role in repairing degenerated cartilage tissue. Accordingly, we observed that UCMSCs significantly promoted the expression of chondrocyte-related genes (*aggrecan*, *Col2*, and *Sox*9) in rat chondrocytes pre-treated with IL-1β, suggesting the protective effect on chondrocytes in vitro (*P* < 0.05; *P* < 0.01; Fig. 3B).

### Umbilical cord mesenchymal stem cells inhibited chondrocyte apoptosis

**Figure 4:**
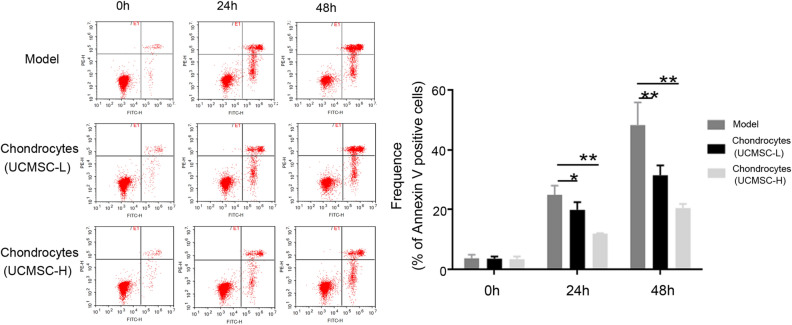
The effect of UCMSCs on the apoptosis of chondrocytes induced by IL-β1. Chondrocytes co-cultured with UCMSCs and IL-β1 were collected to test the rate of chondrocyte apoptosis by flow cytometry. Both high (1 × 10^6^ cells) and low (0.5 × 10^6^ cells) doses of UCMSCs co-cultured with chondrocytes for 24 and 48 h significantly reduced the apoptosis rate of chondrocytes. Model, chondrocytes pre-treated with IL-1β; UCMSC-L, chondrocytes pre-treated with IL-1β co-cultured with a low dose of UCMSCs; UCMSC-H, chondrocytes pre-treated with IL-1β co-cultured with a high dose of UCMSCs, n = 3. *P < 0.05, **P < 0.01 analyzed by unpaired two-tailed t-test after normality and equal variance test using Shapiro–Wilk test and ANOVA plus Brown-Forsythe test in the GraphPad software.

**Figure 5:**
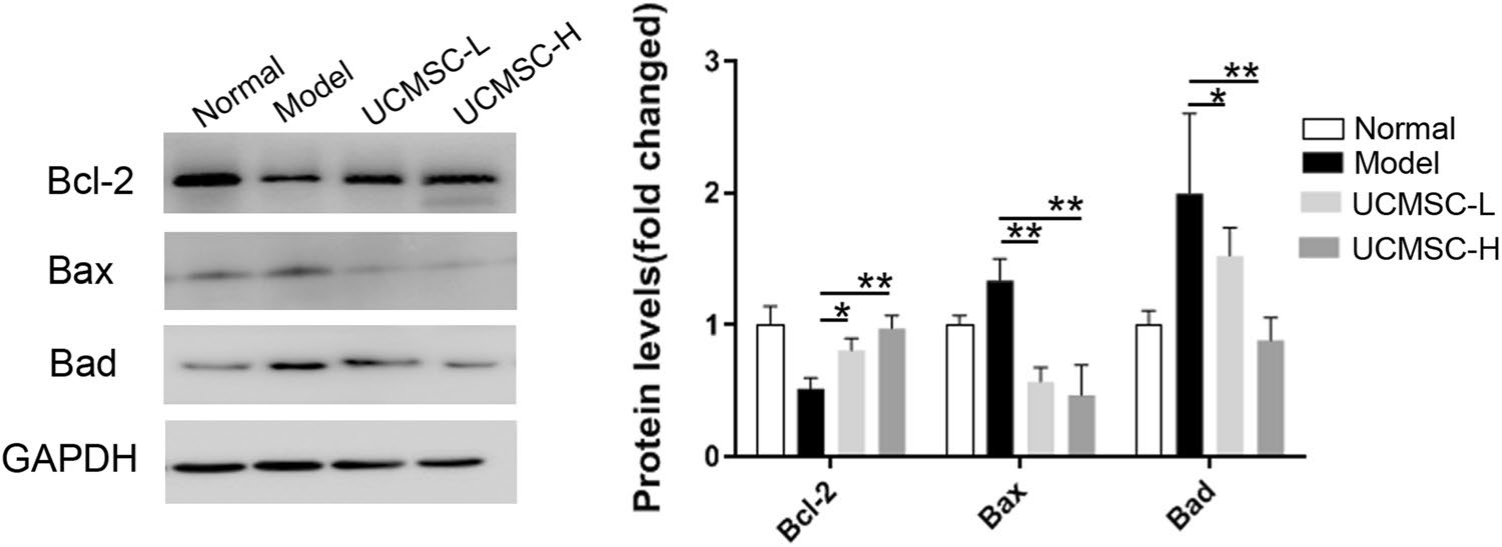
The effect of UCMSCs on apoptosis-related proteins in chondrocytes induced by IL-β1. After co-culture, chondrocytes were collected and used to extract total protein. Cropped gels and western blots show that, compared with the model group, treatment with different doses of UCMSCs significantly upregulated the protein expression levels of Bcl-2. In contrast, they inhibited the expression of Bax and Bad. Full-length blots and gels are presented in Supplementary Fig. 3. Normal, normal group; Model, OA model group; UCMSC-L, Low dose UCMSCs treatment group; UCMSC-H, High dose UCMSCs treatment group, n = 3. *P < 0.05, **P < 0.01 analyzed by unpaired two-tailed t-test after normality and equal variance test using Shapiro–Wilk test and ANOVA plus Brown-Forsythe test in the GraphPad software.

Flow cytometry was used to measure the apoptotic rate of chondrocytes co-cultured with UCMSCs for 24 and 48 h. Our findings indicated that both high and low doses of UCMSCs significantly reduced the apoptosis rate of chondrocytes within 48 h of co-culture (*P* < 0.05, *P* < 0.01; Fig. 4). We performed western blotting to detect the expression of apoptosis-related proteins in chondrocytes co-cultured with UCMSCs. According to the cropped gels and western blotting results, compared to the OA model group, treatment with different doses of UCMSCs significantly upregulated the expression levels of Bcl-2 (*P* < 0.05, *P* < 0.01). In contrast, treatment with UCMSCs inhibited the expression of Bax and Bad (*P* < 0.05, *P* < 0.01) (Fig. 5). Taken together, our results indicated that UCMSCs can reduce the apoptotic rate of chondrocytes.

### Anti-inflammatory role of umbilical cord mesenchymal stem cells in an osteoarthritis model

**Figure 6:**
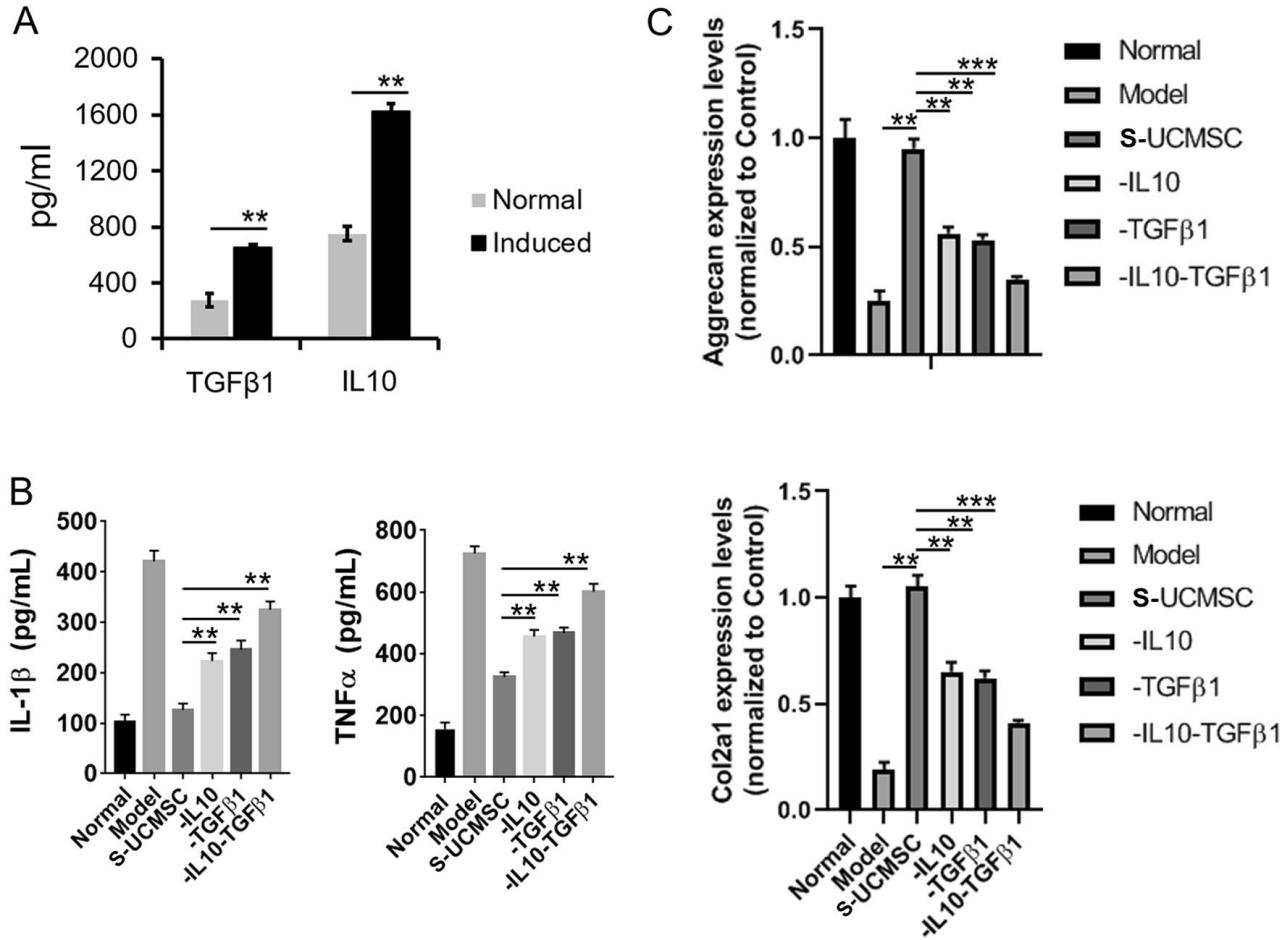
UCMSCs inhibited the inflammatory process of OA by secreting IL-10 and TGF-β1. (**A**) After co-culture with chondrocyte, the expression levels of IL-10 and TGF-β1 in UCMSCs increased significantly. Normal, normal cultured UCMSCs; Induced, chondrocyte co-cultured with UCMSCs. (**B**) Injection of the supernatant from co-cultured UCMSCs into OA model rats reduced the expression levels of IL-1β and TNF-α in the joint cavity fluid of rats. (**C**) Transplantation of UCMSC supernatant into OA model rats increased the expression levels of *aggrecan* and *Col2a1* genes in chondrocytes. Normal, normal group; Model, OA model group; S-UCMSC, supernatant of co-cultured UCMSCs without removing IL10 or TGF-β1 transplantation group; − IL10, supernatant of co-cultured UCMSCs with IL-10 removed; − TGF-β1, supernatant of co-cultured UCMSCs with TGF-β1 removed; − IL10–TGF-β1, supernatant of co-cultured UCMSCs with IL-10 and TGF-β1 removed. n = 6. **P < 0.01, ***P < 0.001 analyzed by One-way ANOVA with Bonferroni test after normality and equal variance test using Shapiro–Wilk test and ANOVA plus Brown–Forsythe test in the GraphPad software.

ELISAs were used to determine the expression levels of IL-10 and TGF-β1 in UCMSCs co-cultured with chondrocytes. Our results showed that IL-10 and TGF-β1 secretion by UCMSCs significantly increased following chondrocyte stimulation (*P* < 0.01; Fig. 6A). In vivo studies have shown that the supernatant of co-cultured UCMSCs can also reduce the levels of IL1-β and TNF-α in the joint cavity fluid (*P* < 0.01; Fig. 6B), while increasing the expression levels of the aggrecan and *Col2a1* chondrocyte genes (*P* < 0.01; Fig. 6C). Notably, the removal of IL-10 or TGF-β1 from the culture supernatant significantly inhibited these effect (*P* < 0.01). Furthermore, simultaneous depletion of IL-10 and TGF-β1 markedly inhibited the effects of UCMSC-derived supernatants (*P* < 0.001). This suggested that UCMSC-secreted IL-10 and TGF-β1 play important roles in inhibiting joint cavity inflammation and protecting chondrocytes in OA.

## Discussion

OA is a prevalent progressive inflammatory disease that commonly affects older adults and may ultimately result in the need for total knee arthroplasty. However, current therapies only aim to manage the symptoms, rather than repair the cartilage or cure the disease. MSCs possess immune-regulating, damage-repairing, and inflammation-controlling properties^25,26^; therefore, their transplantation has emerged as a promising treatment approach for OA.

In this study, we conducted an in vivo experiment to investigate the therapeutic effect of UCMSCs in OA by transplanting them into the joint cavities of rats using an OA model. Our results, which included X-ray analysis, visual observation, histopathological examination, and ELISAs, indicated that the transplantation of UCMSCs significantly improved articular surface damage and repaired the joint structure in rats with OA. These findings were consistent with those of previous literature studies. For instance, Xing et al. administered hUCMSCs to a rat OA model as a single dose of 10^6^ cells in 100 μL hyaluronic acid after surgical removal of the medial meniscus. Their results suggested that a single injection of hUCMSCs temporarily slowed the progression of cartilage degeneration in rats with OA, but did not inhibit OA progression for an extended period^24^. Based on these findings, we administered three consecutive injections of 2 × 10^6^ UCMSCs in the present study, which produced a more favorable therapeutic outcome for OA. However, for subsequent clinical studies, additional parameters, including the optimal dose, frequency of administration, and long-term safety of UCMSCs require further verification.

MSC-based OA therapy mainly focuses on the ability of MSCs to regenerate damaged cartilage and reduce persistent inflammation in the joints; however, the precise molecular mechanism remains unclear. Therefore, we conducted in vitro co-culture assays and in vivo injections of UCMSC-derived supernatants to investigate the molecular mechanisms of action of UCMSCs in OA therapy. Our results indicated that co-culturing UCMSCs with chondrocytes promoted the expression of aggrecan, Col2, and Sox9 in damaged chondrocytes induced by IL1-β and reduced chondrocyte apoptosis. We also observed increased IL-10 and TGF-β1 expression levels in UCMSCs after co-culture. Additionally, our in vivo animal experiments demonstrated that the administration of the supernatant from UCMSCs reduced the levels of IL1-β and TNF-α in joint cavity fluid, increased aggrecan and Col2 expression levels in chondrocytes, and exerted similar inflammation-inhibiting and chondrocyte-protective effects as UCMSC transplantation. The depletion of IL-10 and TGF-β1 by immunoprecipitation significantly repressed the therapeutic effect of the UCMSC supernatants, indicating the involvement of these two factors in the effects of UCMSCs on OA repair. These results support the notion that UCMSCs have immunomodulatory effects and promote tissue repair via a paracrine action^27^. However, the exact mechanisms underlying the effects of UCMSCs and their secreted factors during OA therapy require further studies.

In conclusion, our study demonstrated that UCMSC transplantation significantly reduced joint tissue damage and inflammation, protected the joint structure, and improved joint function in a rat model of OA. The mechanism by which UCMSCs accomplished this appears to be through inhibiting inflammation, reducing chondrocyte apoptosis, protecting chondrocytes, and promoting joint tissue repair via the secretion of IL-10 and TGF-β1.

## Methods

### Isolation and culture of umbilical cord mesenchymal stem cells

UCMSCs were provided by S-Evans Biosciences (Hangzhou, China) and were characterized as previously described^28^. Briefly, an umbilical cord obtained from a healthy full-term newborn was cut into small sections of approximately 2 cm. The newborn was not directly involved in the study. Subsequently, Wharton’s jelly was peeled off, cut into small pieces of 1 mm^6^, and centrifuged at 2000 rpm for 3 min. Approximately 1 g of tissue was spread on the bottom of a T75 bottle using a cell scraper for primary adherent cultures. When cells reached 80–90% confluence, 0.25% trypsin–EDTA (Gibco, Carlsbad, CA, USA) was used to digest and passage the cells. The identification of UCMSCs at passage 3 was accomplished by implementing morphological examination, detecting the expression of surface markers, and evaluating mesenchymal lineage differentiation. UCMSCs at passage 5 were used in subsequent experiments.

### Establishment of the rat model of osteoarthritis

Six-to-eight-week-old male Sprague–Dawley rats (weighing 200 ± 20 g) were purchased from Shanghai SLAC Laboratory Animal Co., Ltd. and fed in accordance with the guidelines of the National Institutes of Health for the Care and Use of Laboratory Animals. All experimental procedures were performed in accordance with the ethical guidelines of the Basel Declaration and the International Council for the Science of Laboratory Animals and were approved by the Institutional Animal Care and Use Committee, Zhejiang University (No. 18021). After excluding rats with the largest and smallest weights, the remaining rats were anesthetized by intraperitoneal injection of 3% sodium pentobarbital (0.2 mL/100 g). Subsequently, the rats were laid on their sides with their bodies stretched to disinfect their right knees with ethanol, after which100 µL of 25 mg/mL iodoacetic acid (A502826, Sangon Biotech, Shanghai, China) was administered to the knee joint to induce OA.

### Animal experiments

To investigate the effect of UCMSC transplantation on rats with OA, a total of 24 rats were randomly divided into normal, OA model, vehicle and UCMSC treatment groups. 2 × 10^6^ UCMSCs suspended in 50 μL of normal saline were infused through the right knee joint cavity in the UCMSC treatment group (n = 6) one week after the establishment of the OA model for 3 consecutive times, once per week. In vehicle group, OA rats (n = 6) were injected with the same dose of saline 3 consecutive times, once per week. OA model group rats (n = 6) were not treated with saline or UCMSCs. Normal group rats were normally fed without any treatment and housed for the same length of time to other groups. One week after the last treatment, the animals were sacrificed by an overdose of pentobarbital (150 mg/kg) intraperitoneally. Joint samples and joint cavity fluid were collected for follow-up observation and evaluation.

We further carried out an in vivo articular cavity administration experiment with UCMSC supernatants to study the mechanism whereby IL-10 and TGF-β1 participate in the process of the UCMSC-induced inhibition of OA inflammation. First, in vitro IL-10 or TGF-β1 depletion was performed as described previously^29^. Briefly, the culture medium of UCMSCs co-cultured with rat chondrocytes and 10 ng/mL IL-1β was incubated with anti-IL-10 (ARC9102, ThermoFisher Scientific, MA, USA) or anti-TGF-β1 (MA5-16949, ThermoFisher Scientific) antibodies overnight with stirring at 4 °C. After the addition of protein A-conjugated Sepharose beads (ab193256, Abcam, Cambridge, UK), the samples were incubated at 4 °C for 4 h and then centrifuged at 10,000×*g* for 5 min. The supernatant was collected and used for intra-articular injection in OA rats. A total of 36 rats were randomly divided into six groups: normal, OA model, supernatant of co-cultured UCMSC without removing IL10 or TGF-β1 transplantation group, supernatant of co-cultured UCMSC with IL-10 removed, supernatant of co-cultured UCMSC with TGF-β1 removed, supernatant of co-cultured UCMSC with IL-10 and TGF-β1 removed (n = 6). 50 μL supernatant was infused through the right knee joint cavity in OA model rats, 3 consecutive times, once per week. Normal group rats were normally fed without any treatment and housed for the same length of time to other groups. One week after the last treatment, the animals were sacrificed using an intraperitoneal overdose of pentobarbital (150 mg/kg). Joint samples and joint cavity fluid were collected for follow-up observation and evaluation.

### X-ray imaging and Pelletier score of knee joints

X-ray imaging was performed to examine the structure of the knee joint one week after the third UCMSC transplantation. According to the Kellgren-Lawrence classification standard, scores were calculated based on the size of the knee joint cavity, bone sclerosis, and osteophyte (OP) formation. There was no joint space narrowing (JSN) for grade 0, slight JSN and OP formation for grade 1, mild JSN and OP for grade 2, moderate JSN and OP for grade 3, and severe JSN and OP for grade 4.

### Pathological examination of knee joints

Knee joint tissues were fixed with 4% paraformaldehyde (E672002, Sangon Biotech) at 4 °C for 48 h and then sent to Dian Diagnosis (Hangzhou, China) for decalcification, paraffin sectioning, H&E staining (E607318, Sangon Biotech), and Safranin O staining (A600815, Sangon Biotech). We used a microscope to observe the severity of arthritis and score it in accordance with the OARSI standards, as follows: 0 points, the cartilage surface was flat, and the cartilage was intact; 1 point, there was uneven superficial fiber formation on the cartilage surface; 2 points, the cartilage surface was discontinuous, accompanied by cell proliferation, and metachromatic substances were increased in layers II–III; 3 points, chondrocytes had spread to layer III or the cartilage surface was eroded; 4 points, cartilage erosion worsened, and articular cartilage was damaged; 5 points, the articular cartilage had been exfoliated; and 6 points, the joints were deformed.

### Co-culture of umbilical cord mesenchymal stem cells and chondrocytes in rats

Sprague–Dawley rats (n = 3) aged 3–4 weeks were sacrificed by an overdose of pentobarbital (150 mg/kg) intraperitoneally, and cartilage tissues from the surface of the knee joint femur and tibial plateau were separated under aseptic conditions. The collected cartilage was cut into small pieces (< 1 mm^3^) and digested with 0.25% trypsin (NC1004.1, youcon, China) at 37 °C for 30 min. The cartilage pieces were then digested with 0.2% type II collagenase at 37 °C for 4 h, while shakieng once every 30 min. The cells were filtered using a 70 μm filter (Falcon, BD Biosciences, CA, USA) before washing with phosphate-buffered saline (PBS; E607008, Sangon Biotech). They were then resuspended in DMEM-F12 medium (11320033, gbico, MA, USA) supplemented with 10% fetal bovine serum (E600001, Sangon Biotech) and cultured at 37 °C and 5% CO_2_ for 8–10 days. Cells were passaged when the density of chondrocytes reached 80–90%. Chondrocytes at passage 3 were treated with10 ng/mL IL-1β for 48 h to establish an in vitro injury model and were then washed with PBS before use in further experiments. The cells were then separated into 4 groups: Normal (normal cultured rat chondrocytes), model (rat chondrocytes pre-treated with 10 ng/mL IL-1β), UCMSC-L (rat chondrocytes pre-treated by10 ng/mL IL-1β were co-cultured with 0.5 × 10^6^ UCMSCs), and UCMSC-H (rat chondrocytes pre-treated by 10 ng/mL IL-1β were co-cultured with 1 × 10^6^ UCMSCs). Chondrocytes (1 × 10^6^) and UCMSCs were co-cultured in a transwell chamber (Millipore, MA, USA) for 24 h and 48 h. Chondrocytes were collected for gene expression and apoptosis analyses. In a parallel experiment, after co-culturing 1 × 10^6^ UCMSCs and 1 × 10^6^ rat chondrocytes with 10 ng/mL IL-1β for 48 h, UCMSCs were placed in 2 mL of serum-free medium for another 24 h. The culture media were then collected and passed through a 0.22 µm filter to generate the supernatant for further ELISA and in vivo experiments.

### Detection of gene expression

Cell samples were collected from each group after co-culture, and RNA was isolated according to the manufacturer's instructions (15596026, Invitrogen, CA, USA). Two-step reverse transcription-polymerase chain reaction (PCR) was performed to determine the expression levels of *aggrecan*, *Col2*, *Sox9*, and *Gapdh* genes. The first PCR was as follows: 95 °C for 4 min, followed by 25 cycles at 95 °C for 4 min, 58 °C for 30 s, 72 °C for 40 s, and 72 °C for 10 min. Subsequently, 0.2 μL of the PCR product was used as the template for the second PCR. The second PCR was performed as follows: 95 °C for 4 min, followed by 35 cycles at 95 °C for 4 min, 65 °C for 30 s, and 72 °C for 10 min. The primers used are listed in Supplementary Table 1.

### Flow cytometry

Collected cells were centrifuged at 1000×*g* for 5 min, and the cell pellet was resuspended in 195 μL of annexin V-FITC binding solution. Subsequently, 5 μL of annexin V-FITC (556419, BD Pharmingen) was added to the cells before adding 10 μL of propidium iodide. After mixing well, the mixture was incubated in the dark at 25 °C for 15 min. Apoptosis of cells was detected by flow cytometry (FACS VERSE, BD Biosciences).

### Detection of apoptosis-related proteins

Total cell protein was extracted from cell lysates according to the manufacturer's instructions (P0013, Beyotime, Shanghai, China). The protein concentration was evaluated using a BCA kit (P0011, Beyotime). Protein samples were separated by 12% SDS-PAGE and transferred to polyvinylidene fluoride (PVDF) membranes. The membranes were then trimmed according to the estimated molecular weight of the target protein for subsequent antibody incubation to reduce the amount of antibody required. After incubation in 2% BSA (ST025, Beyotime) at 25 °C for 1 h, the membranes were incubated overnight at 4 °C with primary antibodies, including rabbit anti-Bcl-2 (12789-1-AP, ProteinTech, IL, USA; 1:1000), anti-Bax (AF0057, Beyotime; 1:500), anti-Bad (AF1009, Beyotime; 1:500), and anti-GAPDH (AF1186, Beyotime; 1:2000). After washing with PBST (C520004, Sangon Biotech), the membranes were incubated with goat anti-rabbit IgG secondary antibodies (A0208, Beyotime; 1:1000) at 25 °C for 1 h. Protein bands were visualized using a gel imaging system and analyzed using ImageJ software (National Institutes of Health, MD, USA; v1.8.0).

### Enzyme-linked immunosorbent assay

Joint cavity fluid was collected by rinsing the joint cavity twice with 100 μL of PBS each time. The joint cavity fluid was centrifuged at 150×*g* for 10 min at 4 °C, and the supernatant was collected. The supernatants of the co-cultured cells from the different treatment groups were also collected. ELISA kits were used to determine the levels of IL-1β (EK0392, Boster, China), TNF-α (EK0525, Boster), MMP13 (EK0468, Boster), IL-10 (EK0416, Boster), and TGF-β1 (EK0513, Boster) in the supernatant.

### Statistical analysis

All data were analyzed using GraphPad Prism 6.0 (GraphPad software, CA, USA) and are presented as the mean ± standard deviation^30^. The data were passed by normality and equal variance test using Shapiro–Wilk test and ANOVA plus Brown-Forsythe test in the GraphPad software. Then unpaired two-tailed t-test in was used to compare the differences between two groups. One-way ANOVA with Bonferroni test was performed for multiple group comparison. *P < 0.05 indicated a significant difference.

### Ethics statement

The study was reported in accordance with ARRIVE guidelines. All experimental procedures were performed according to the Basel Declaration and the International Council for the Science of Laboratory Animals (ICLAS) ethical guidelines and approved by the Institutional Animal Care and Use Committee of Zhejiang University (No. 18021).

### Supplementary Information

Supplementary Figure 1.

Supplementary Figure 2.

Supplementary Figure 3.

Supplementary Table 1.

## Data Availability

The data of this study can be provided by the corresponding author upon reasonable request.
