# Supplementary material for: Umbilical cord mesenchymal stem cells relieve osteoarthritis in rats through immunoregulation and inhibition of chondrocyte apoptosis
Source: Sci Rep. 2023 Sep 11;13:14975. doi: 10.1038/s41598-023-42349-x (PMC10495383; doi:10.1038/s41598-023-42349-x)
Supplement: Supplementary file 1 — Supplementary Figure 1. [file 41598_2023_42349_MOESM1_ESM.pdf]

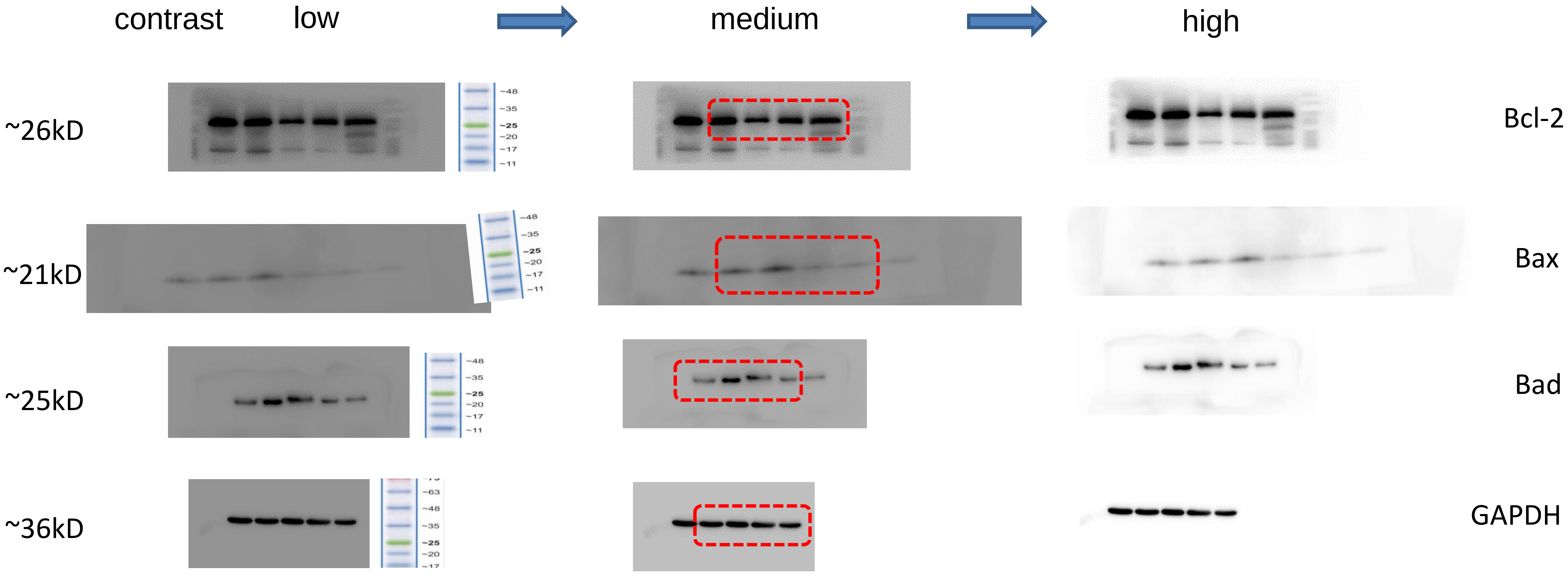

Red dotted box indicated WB area shown in the manuscript. The transferred western blot membrane is trimmed according to the estimated molecular weight of the target protein for subsequent antibody incubation to save the amount of antibody.
