# Supplementary figures and images for: Umbilical cord mesenchymal stem cells relieve osteoarthritis in rats through immunoregulation and inhibition of chondrocyte apoptosis
Source: Sci Rep. 2023 Sep 11;13:14975. doi: 10.1038/s41598-023-42349-x (PMC10495383; doi:10.1038/s41598-023-42349-x)

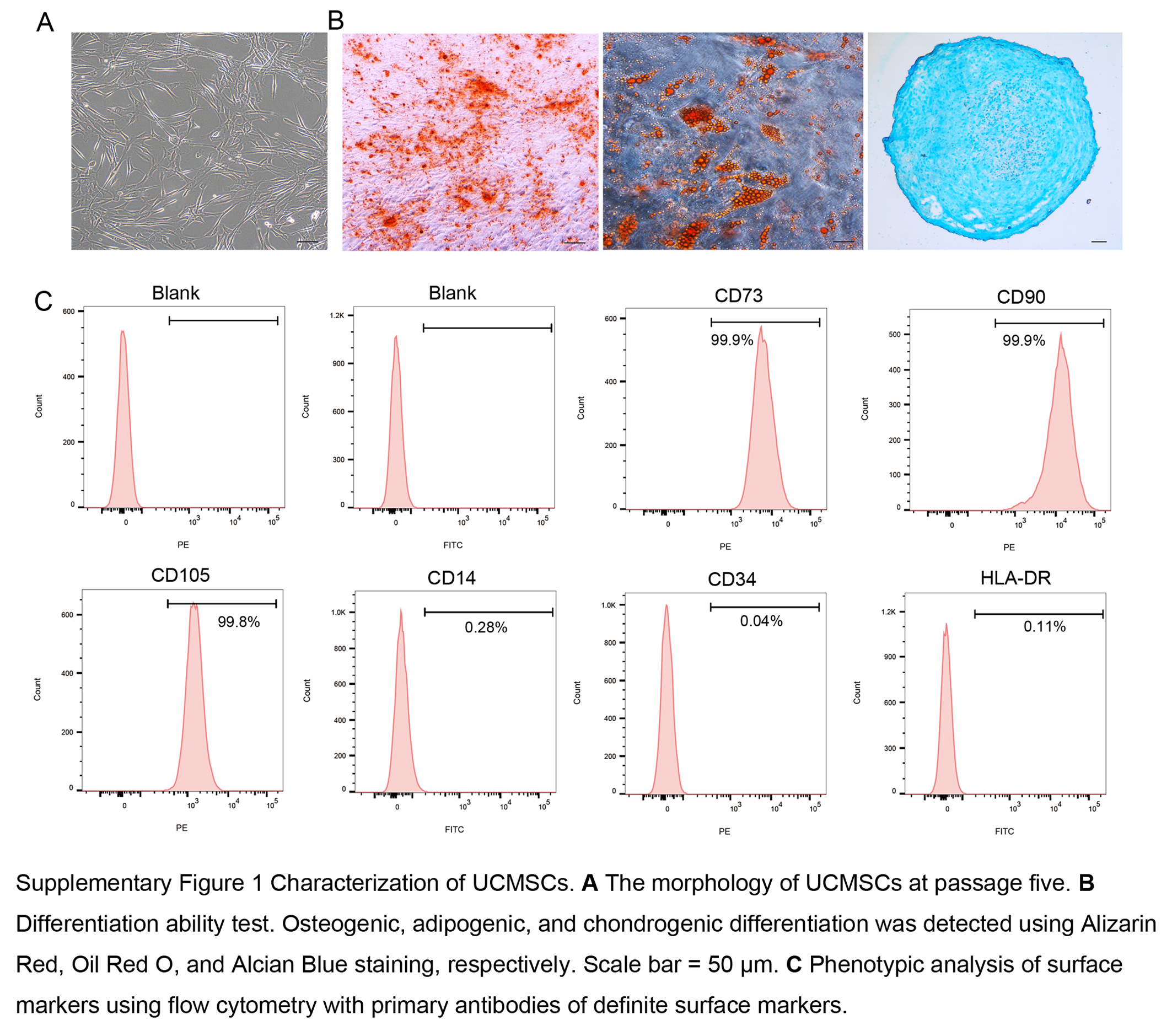

Supplement: Supplementary file 2 — Supplementary Figure 2. [file 41598_2023_42349_MOESM2_ESM.tif]

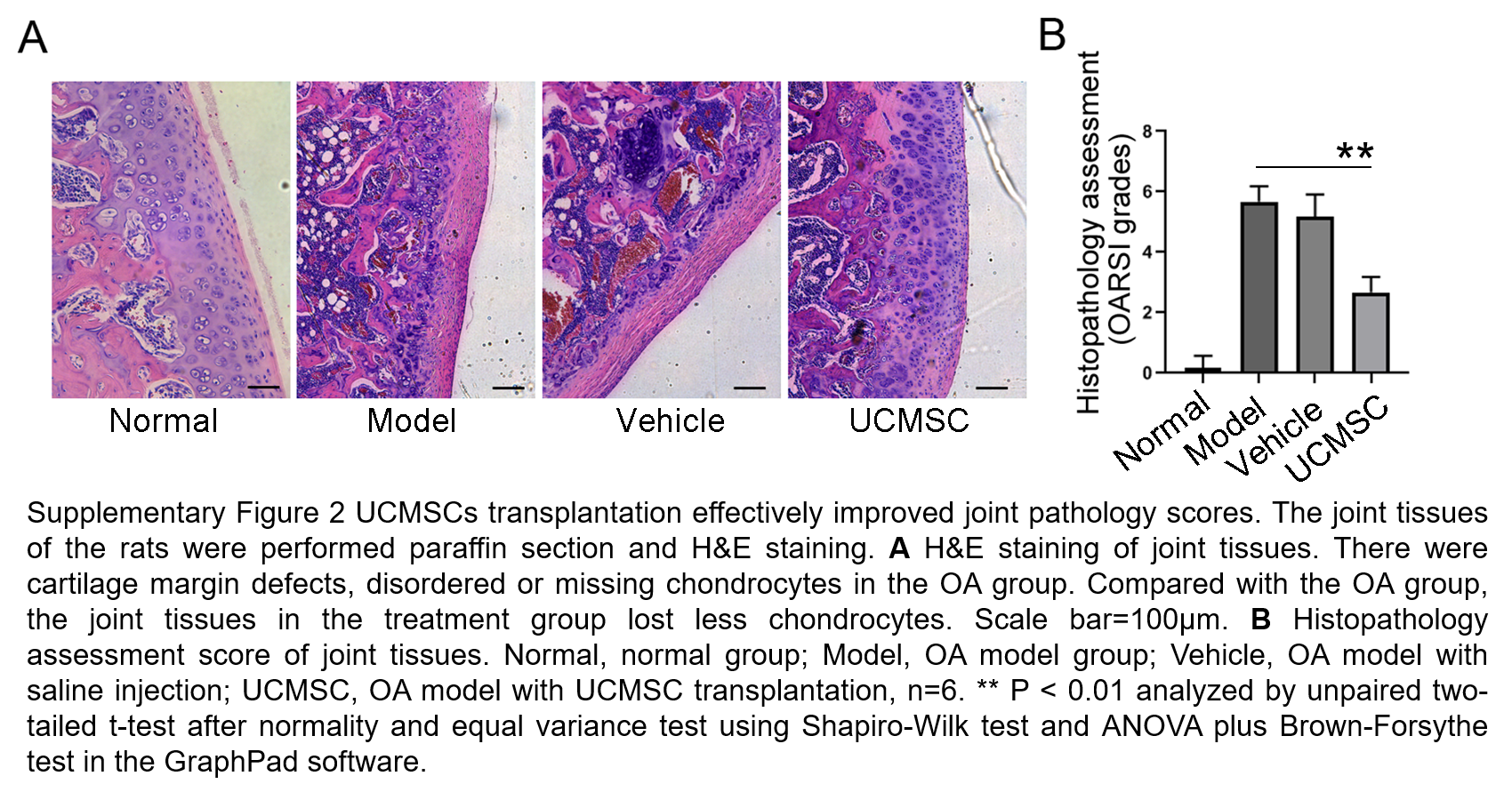

Supplement: Supplementary file 3 — Supplementary Figure 3. [file 41598_2023_42349_MOESM3_ESM.tif]
