# Supplementary material for: Umbilical cord mesenchymal stem cells relieve osteoarthritis in rats through immunoregulation and inhibition of chondrocyte apoptosis
Source: Sci Rep. 2023 Sep 11;13:14975. doi: 10.1038/s41598-023-42349-x (PMC10495383; doi:10.1038/s41598-023-42349-x)
Supplement: Supplementary file 4 — Supplementary Table 1. [file 41598_2023_42349_MOESM4_ESM.docx]

Table 1 List of primers for chondrocyte-related genes

| Gene name | Forward | Revere |
| --- | --- | --- |
| *Aggrecan* | TGAGCGGCAGCACTTTGAC | TGAGTACAGGAGGCTTGAGG |
| *Col2* | TCAGGAATTTGGTGTGGACAT | CCGGACTGTGAGGTTAGGATAG |
| *Sox9* | ATGAAGATGACCGACGAGCA | CAGTCGTAGCCTTTGAGCAC |
| *GAPDH* | GGCACAGTCAAGGCTGAGAATG | ATGGTGGTGAAGACGCCAGTA |
